# Stress alters hypothalamic gene expression in adolescent male Golden hamsters

**DOI:** 10.1111/jne.70067

**Published:** 2025-07-14

**Authors:** Kevin M. Moran, Tyler M. Milewski, James P. Curley, Yvon Delville

**Affiliations:** ^1^ Joint Graduate Program in Toxicology, Rutgers The State University of New Jersey Piscataway New Jersey USA; ^2^ Psychology Department The University of Texas at Austin Austin Texas USA

**Keywords:** obesity, puberty, RNAseq, social stress, WGCNA

## Abstract

In Golden hamsters (*Mesocricetus auratus*), a two‐week exposure to chronic social stress in adolescence causes acceleration of agonistic behavior, enhanced adult aggression, impaired waiting impulsivity, and higher food intake, body fat, and long‐term increased body weight. In adult rodents, stress is accompanied by widespread alterations in gene expression in the brain. As stress is a potent modulator of both gene expression and behavior, the present research investigated possible mechanistic‐related transcriptomic changes in the lateral, dorsomedial, and arcuate nucleus of the hypothalamus caused by adolescent stress using RNA Tag‐sequencing, as these areas are involved in the regulation of metabolic and motivated behaviors. In each region, there were approximately 250 genes with higher expression compared to controls and 250 genes with lower expression. Many of the most significantly affected genes have been associated with metabolism and sex hormone function. For example, in the lateral hypothalamus, melanocortin 3 receptor, growth hormone releasing factor, both involved in metabolic processes, and neuropeptide VF precursor, involved in growth hormone inhibitory hormone production, were among the most increased in expression in stressed subjects. In the dorsomedial hypothalamus, neuropeptide W, involved in feeding cessation, was significantly decreased in expression in stressed animals. Across both regions, G‐protein coupled receptor 50, involved in thermoregulation, sleep, and sex‐related mood disorders, was significantly altered, but in opposite directions. In the arcuate nucleus, a number of blood brain barrier‐ and inflammation‐related genes were altered as well. Furthermore, there were consistent patterns of genetic ensembles identified through gene ontology analysis and weighted gene correlation network analysis that were altered across each region. Many of these involved roles in RNA processing, DNA methylation, myelination, and synaptic organization. These findings reinforce prior behavioral, hormonal, and metabolic changes observed in this developmental model, and help guide future directions of research related to the negative consequences of early life stress.

## 1 INTRODUCTION

Stress is an unavoidable circumstance to which species have developed various coping strategies, such as conditioned avoidance or learned helplessness.^1^, ^2^, ^3^, ^4^ These behaviors are malleable and related to prior experience and time of exposure, and can be adaptive or maladaptive depending on the following life circumstances. It is well known that prior stressful experiences during childhood and adolescence increase the likelihood of adult behavior, mood, and health disorders.^5^, ^6^, ^7^, ^8^ Additionally, in many humans, chronic stress tends to cause increased consumption of high‐calorie foods, though in a smaller set of individuals, it promotes anorexic behaviors,^9^, ^10^, ^11^ which is also related to fetal or childhood experiences. For example, individuals that experienced abuse or higher numbers of adverse childhood events, such as domestic violence at home, have a higher likelihood of being overweight as adults.^8^, ^12^, ^13^, ^14^

One well‐characterized example of this phenomenon in an animal model is the differential response to social defeat stress in adult and adolescent Golden hamsters (*Mesocricetus auratus*). In adult Golden hamsters, a single loss causes conditioned defeat behaviors, in which losers/subordinate individuals show reduced aggression to conspecifics and likely lose any altercations in the future.^3^, ^15^ Adult social defeat causes widespread *c‐Fos* expression throughout the brain, including the hypothalamus,^16^ and persists through 7 days of chronic defeat in this species.^17^ In adults, a single defeat also induces differential expression of over 500 gene transcripts in the basolateral amygdala.^18^ Increasing the expression of genes in the medial prefrontal cortex via histone deacetylase inhibition also enhances social defeat behaviors in adults, while inhibition of acetylation reduces them.^19^

Conversely, chronic exposure to social stress in adolescence leads to different behavioral outcomes in male, but not necessarily female, Golden hamsters.^20^, ^21^ Instead of presenting conditioned defeat, previously stressed individuals are more likely to attack smaller opponents once they reach adulthood.^21^, ^22^ This stressed phenotype includes an acceleration of the maturation of agonistic behavior from play fighting to aggression,^21^ selective avoidance and enhanced risk assessment behaviors,^23^ altered motor impulsivity,^24^, ^25^ accelerated weight gain, higher food intake, enhanced body fat, long‐term increased body weight, and altered food‐related preference behaviors.^26^, ^27^ Additionally, adolescent stress alters innervation in a variety of systems involved in these behavioral and metabolic outcomes, including vasopressin, serotonin, and orexin.^22^, ^28^ Clearly, a large number of behavioral and homeostatic‐regulatory systems are impacted by adolescent stress.

In the present study, we used a global transcriptomic approach to identify potential gene targets in the hypothalamus that may be expressed due to chronic social stress exposure in adolescent male hamsters. We focused on three hypothalamic regions for analysis: lateral hypothalamus (LH), dorsomedial hypothalamus (DMH), and arcuate nucleus of the hypothalamus (ARC). We chose these regions as they are reciprocally connected and contribute to a variety of behaviors and homeostatic mechanisms that are altered in the adolescent stressed hamster model.^29^, ^30^ The ARC and LH exert major influence over food intake and body weight, with the ARC monitoring internal energy states and sending signals to the LH, which then act downstream to influence appetitive and ingestive behaviors.^31^, ^32^ Specific cell populations in the LH, such as orexin/hypocretin, also influence reward‐related behaviors, both of which are altered by stress in adolescent hamsters.^24^, ^28^, ^33^ The DMH is also involved in appetitive and homeostatic behaviors,^34^, ^35^ as well as sex hormone production,^36^ which is downregulated after stress exposure in male adolescent Golden hamsters.^37^

We predicted a number of genes or gene modules related to aggressive behavior, reward and decision making, metabolic processes, and development‐related genes to be altered in stressed subjects. Specifically, given the phenotypic changes pertaining to food intake and metabolism that are observed in socially defeated adolescent hamsters, we hypothesized enhanced transcriptional changes to orexigenic processes and decreased expression related to anorexigenic processes.

## 2 METHODS

### 2.1 Animals and housing

Golden hamsters (*M. auratus*) were bred in the laboratory from a colony initially obtained from Harlan Sprague–Dawley (Aura strain, Indianapolis, IN) and kept at the Animal Resource Center, an AAALAC‐accredited facility. All procedures were consistent with NIH guidelines and approved by the IACUC from the University of Texas at Austin. Each litter was culled to six pups, including males and females, on postnatal day 7 (P7). On P25, all male animals were weaned and single‐housed in Plexiglas cages (19 W x 43.2D x 26.5H cm) enriched with cotton pads for nesting materials and food piles, as this species hoards food in natural and laboratory environments.^38^, ^39^

The animals were kept in a reverse light cycle (14L–10D, lights off at 10:00 a.m.) and received food [Prolab RMH 1800 5LL2 rodent diet, Lab Supply, Dallas‐Fort Worth, TX (3.15 kcal/g; 21.2% protein, 13.7% fat, 65.1% carbohydrate)] and water ad libitum. For habituation prior to and during behavioral tests, diet was supplemented with banana‐flavored food pellets [Dustless Precision Pellets®, 45 mg, Primate Purified Diet, Banana flavor, Bio Serv, Flemington, NJ (3.45 kcal/g; 20.2% protein, 6.3% fat, 52% carbohydrate)] as part of conditioning protocols. All behavioral procedures occurred between 11:00 a.m. and 3:00 p.m., Zeitgeber time 15 to 18–1 to 4 h into the dark/active cycle. Starting 2 days before the stress and control procedures, hamsters were weighed and had all food removed from their home cages and weighed, then “topped off” to 80 g. Weighing always occurred prior to stress or control procedures. This process was repeated every 2 days until the end of the stress period. Similar to other studies,^26^, ^40^, ^41^ we analyzed food intake as cumulative food eaten over time. In this experiment we focus on male hamsters, as adolescent social stress exposure in females does not chronically alter cortisol levels or accelerate the development of agonistic behavior as it does in males, suggesting adolescent females habituate to repeated social stress.^20^

### 2.2 Social stress

**FIGURE 1:**
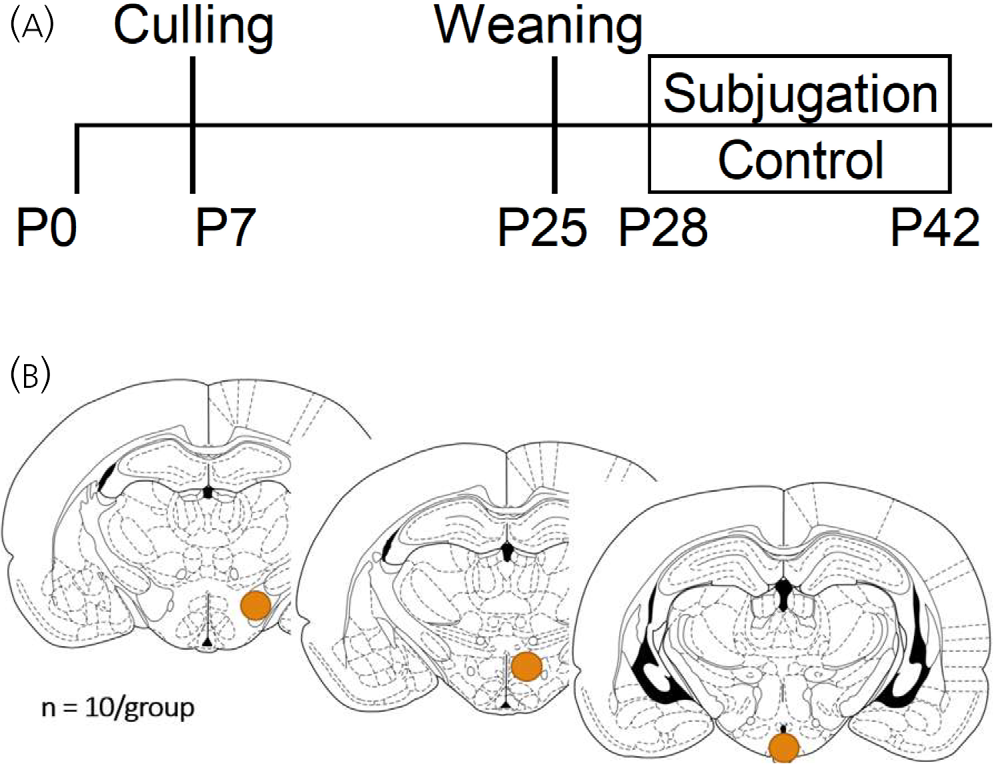
Experimental timeline and regions of interest. (A) Diagram of experimental timeline. Postnatal day 0 (P0) represents birth. Litters were culled to 6 hamsters on P7 and weaned to individual housing on P25. Social Stress or Control procedure exposure occurred from P28 to P42. Animals were sacrificed on P43. (B) Depiction of regions punched for Tag‐seq. Left to right, lateral hypothalamus, dorsomedial hypothalamus, and arcuate nucleus of the hypothalamus. Punches were taken bilaterally. Brain drawings here are from the Paxinos and Watson Rat Brain Atlas,^43^ though a hamster atlas^44^ was used to guide tissue collection.

From P28 to P42, hamsters underwent either daily social stress or control handling procedures. This period corresponds to early puberty in this species.^42^ Experimental animals (Stressed) were placed in the home cage of an adult, experienced fighter male for 20 min.^21^, ^22^ To control for handling and new‐cage exposure, control subjects (Control) were placed into an empty, clean cage for 20 min (Figure 1A). Adolescents were rotated through seven adult males, such that they did not meet the same male more than twice and did not form a stable relationship with any of them. While control animals eventually habituate to this process, the subjugated animals do not and continue to show a stress response at P42.^45^ All animals were checked for injuries after resident‐intruder interactions, though none occurred as fights between Golden hamsters rarely cause injuries.^46^

During stress, an observer recorded behaviors from the adult resident and the juvenile intruder. These recordings included the number of attacks by the resident, the number of times the resident chased the intruder, the number of times the intruder displayed his tail up (a mildly submissive posture), the number of times the intruder laid on his back without the resident's physical influence (a highly submissive posture), and the number of times the resident flank marked his home cage. On average, Stressed subjects received 0–2 attacks per day, displayed 1–3 tail up behaviors per day, and 0–1 of the other recorded social behaviors were scored, similar to previously reported data.^28^ Prior research has shown little correlation between these behaviors and metabolic metrics,^26^ food conditioned place preference behaviors^27^ or orexin innervation in a variety of brain regions^28^; thus, they were not a major focus of analysis and were primarily recorded to ensure defeat of the intruder.

### 2.3 Tissue collection and RNA extraction

Twenty subjects total were used in this analysis (10 Control, 10 Stressed). Subjects were rapidly decapitated 24 h after final stress or control exposure, and brains were flash frozen on dry ice. Tissue was stored at −70°C until tissue punching. White adipose tissue deposits from mesenteric, epididymal, inguinal, and retroperitoneal areas were extracted and weighed, as described in previous studies.^26^, ^40^ The sum total of these is reported below alongside other recorded metabolic measures.

Brain regions were identified using a Golden Hamster stereotaxic brain atlas.^44^ Tissue was sectioned using a cryostat (Leica Biosystems, Deer Park, IL) at 300 μm, then extracted with a Stoelting 0.75 mm tissue punch (Stoelting, Wood Dale, IL, Cat. No. 57401) centered on regions of interest: lateral hypothalamus (LH) (bregma −2.0 mm), dorsomedial hypothalamus (DMH) (bregma −2.3 mm), and arcuate nucleus of the hypothalamus (ARC) (bregma −2.9 mm) (Figure 1B). Bilateral punches were taken from two sections for each region per animal and pooled for sampling. Samples were homogenized in 100 μL lysis buffer (Thermo Fisher Scientific, Waltham, MA; MagMax Total RNA isolation kit, Cat. No. A27828) with 0.7% beta‐mercaptoethanol by vortexing at 3000 rpm speed for 15–20 s. Lysates were incubated at room temperature for 5 min and stored at −80°C until RNA extraction. Once dissection of all individuals was completed, we proceed to RNA extraction using the KingFisher Flex (Thermo Fisher Scientific, Cat. No. 5400630l) with an additional DNase step to remove DNA contamination added according to the manufacturer's protocol. Samples from each brain region of all animals were extracted in the same batch. RNA quality was determined using RNA 6000 Nano Assay with BioAnalyzer (Agilent Technologies, Santa Clara, CA) and RNA concentration was determined with Quant‐it RNA High Sensitivity assay kit (Thermo Fisher Scientific, Cat. No. Q33140). RNA samples were normalized to 100 ng/μL and stored at −80°C before sequencing.

Samples were submitted to the Genome Sequence and Analysis Facility at the University of Texas at Austin for Tag‐based RNA sequencing (Tag‐seq). This method is an efficient and cost‐effective approach specifically designed to measure abundances of polyadenylated transcripts, yielding highly reliable data for differential gene expression analysis in well‐annotated genomes.^47^, ^48^ It requires very few sequencing reads and is resilient to variation in sample integrity.^49^ While it does not yield complete RNA sequences, including splice variants (i.e., coding and non‐coding RNA), it is suitable for identifying coding genes that are differentially expressed. With these transcriptome profiles, we identified differentially expressed genes (DEGs) between Stressed and Control hamsters. All samples were processed in the same batch throughout the process. Libraries were constructed with a protocol modified from references 47, 48. Reads were sequenced on the NovaSeq 6000 SR100, with minimum reads of 4 million and the target reads per sample of 5 million.

### 2.4 Data analysis

All statistical analyses were completed using R v4.3.2.^50^, ^51^

#### 2.4.1 Metabolic and stress metrics analysis

Welch's *t*‐tests were used to assess pairwise mean group differences in metabolic metrics, which included: body weights, food intake, rate of weight gain, food efficiency (grams of weight gained/grams of food eaten), and fat mass. Welch's *t*‐tests reported as [mean ± standard deviation; *t*(df) *t* value with estimated degrees of freedom; *p* = *p* value with *α* = .05, two‐tailed, *d* = effect size using Cohen's *d*]. Trends (*p* < .1) are also reported and discussed further below.

Behaviors recorded during social stress were correlated with the top 10 most differentially expressed genes (the five genes with the highest positive and negative relative expression, determined by highest absolute log2FC and eFDR <0.05, described below). Only average attacks received by the intruder, tail‐ups displayed by the intruder, and scent markings from the resident were correlated, as the other behaviors occurred with too little frequency for valid correlation. All comparisons were made with Spearman correlation, as gene expression data was not normally distributed, and Holm‐corrected for multiple comparisons.^52^, ^53^, ^54^

#### 2.4.2 Bioinformatics analysis

Raw RNA reads were processed to obtain gene count data by following the TagSeq data processing pipeline provided based on references 47, 48. Briefly, a customized perl script utilizing FASTX‐Toolkits and CUTADAPT 2.8^55^ was used to remove reads with a homo‐polymer run of “A” ≥8 bases and retain reads with a minimum 20 bases while removing PCR duplicates. Processed reads were then mapped to the annotated BCM_Maur_2.0 *M. auratus* genome, which has over 97% mapped reads and 21,616 annotated protein‐coding genes and 10,459 annotated noncoding genes,^56^ using Bowtie2 and STAR.^57^ Differential gene expression analysis was conducted using the Bioconductor package limma.^58^ For each brain region, we conducted principal component analysis filtered gene counts data (filtered out genes with less than 10 counts for each sample) to visually inspect for outliers. No outliers were detected for any region. Filtered read counts were then normalized to account for different library sizes among samples with a voom transform. Differentially expressed genes were identified between Control and Stressed subjects. We adjusted the raw *p*‐value via empirical false discovery rate (eFDR).^59^ To estimate eFDR, we permuted sample IDs 5000 times and obtained a null distribution of *p*‐values. The significance threshold for differentially expressed genes (DEGs) was set as a log 2‐fold change of 1.2 (a 15% difference) with an eFDR of 5% (*p* < .05). We also performed Gene Ontology (GO) analysis to explore differences among identified DEGs within functional modules between groups using the clusterProfiler R package.^60^ Regions were validated by separation in principal component analysis (Figure S1) and the presence of marker genes that are well known to be in these regions (*hcrt* in the LH, *npvf* in the DMH, and *npy* in the ARC).^61^, ^62^, ^63^

#### 2.4.3 Weighted gene co‐expression network analysis (WGCNA)

Weighted gene co‐expression network analysis (WGCNA) was performed using the WGCNA R package.^64^ WGCNA constructs Pearson correlation matrices of RNA expression and clusters highly co‐expressed genes into several modules. WGCNA then examines the association between given experimental factors and module eigengenes (MEs), assigned to colors, which are the first component from the principal component analysis (PCA) for each module. WGCNA further calculates module membership for each gene. Module membership (MM) is measured as the Pearson correlation between the gene expression level and the module eigengene, with an absolute value of module membership close to 1 indicating that the gene is highly connected to other genes in the module. Gene counts were normalized with the DESeq2 package and genes that had low expression counts (<15) in more than 75% of samples were filtered out prior to network construction. Each WGCNA model was constructed in signed hybrid mode. Power was selected by selecting a value with scale‐free topology model fit above 0.8 with a minimal mean connectivity (12 for LH, 10 for DMH, and 6 for ARC). We tested whether each module eigengene was significantly different between groups using linear regression models, which is useful for identifying differential associations of functional modules and has been used in other WGCNA analysis^65^, ^66^ and report modules that are statistically significant between group (*b* = beta coefficient ± standard error; *p* = *p*, with *α* = .05, two‐tailed). We performed GO analysis on these modules to identify functions of independently clustered modules. Finally, we extracted hub genes with very high functional module membership.

## 3 RESULTS

### 3.1 Metabolic metrics

**FIGURE 2:**
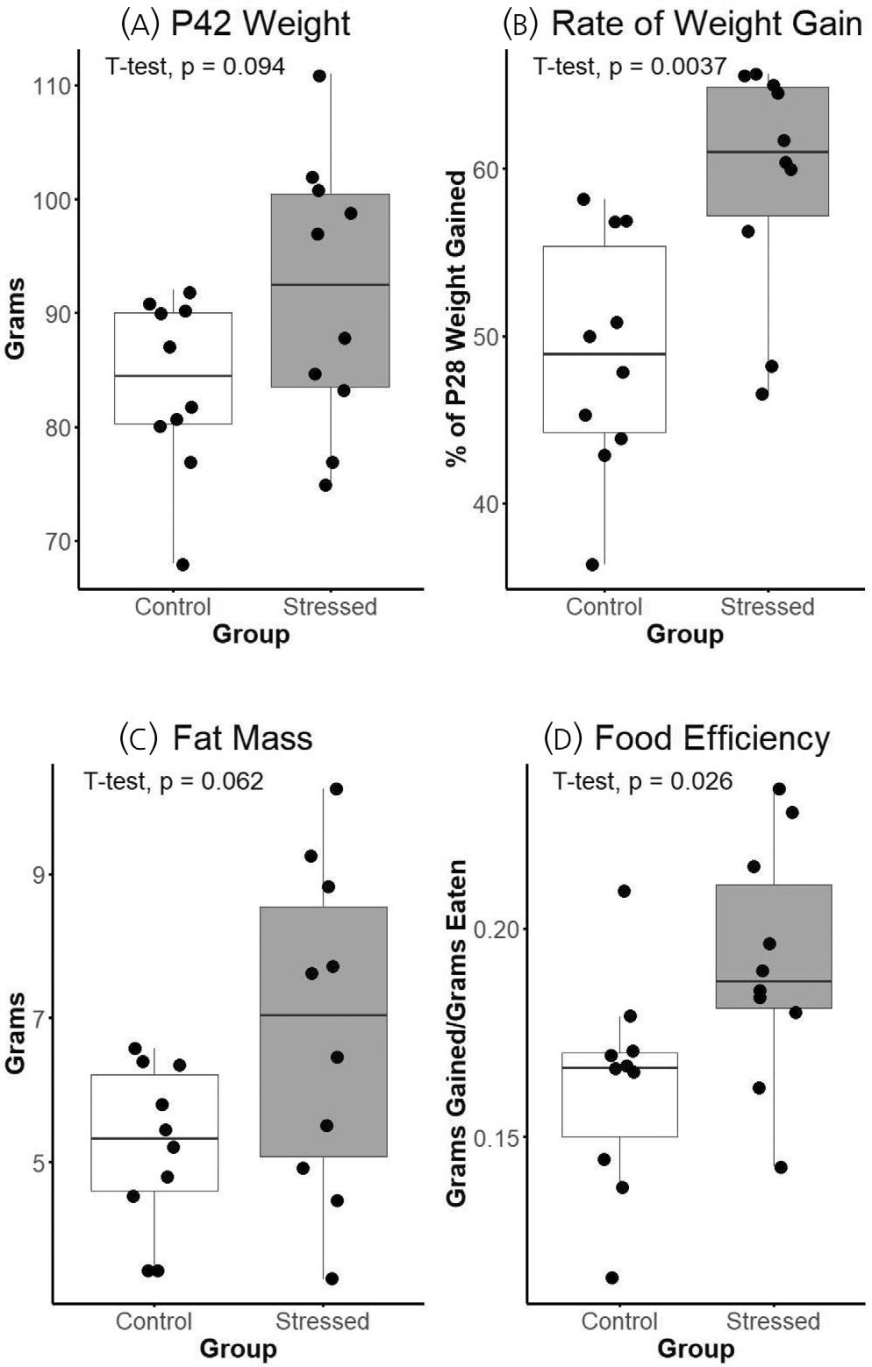
Boxplots of metabolic measures between Stress and Control subjects. (A) Body weight at P42 in grams. (B) Percent of weight at P28 gained by P42. (C) Total mass of white adipose tissue pads collected in grams. (D) Total food efficiency over stress period as grams of weight gained divided by grams of food eaten. Boxplots are depicted with median line and first and third quartile ranges in boxes, with 95% range as whiskers. Points represent the y‐axis value of each subject. Significance results of Welch two‐sample, two‐tailed *t*‐tests are reported for each measure.

There were no group differences in body weight at P28, and Stressed subjects trended heavier at P42 [Control: 83.8 g ± 7.66; Stressed: 91.8 g ± 11.9; *t*(15.35) = 1.78, *p* < .094, *d* = 0.80], with Stressed subjects weighing about 10% more than controls. The rate of weight gain was significantly higher in Stressed subjects [Control: 48.89% ± 7.08; Stressed: 59.38% ± 7.01; *t*(18.00) = 3.33, *p* < .01, *d* = 1.49]. Body fat total at P42 trended higher in Stressed hamsters [Control: 5.21 g ± 1.13; Stressed: 6.83 g ± 2.25; *t*(13.27) = 2.04, *p* = .062, *d* = 0.91]. Despite no group difference in total food intake, Stressed subjects had enhanced food efficiency (gaining more weight per gram of food eaten) [Control: 0.162 ± 0.025; Stressed: 0.191 ± 0.028; *t*(17.75) = 2.42, *p* < .05, *d* = 1.09] (Figure 2).

### 3.2 Stressed hamsters show differential gene expression in hypothalamic nuclei

**FIGURE 3:**
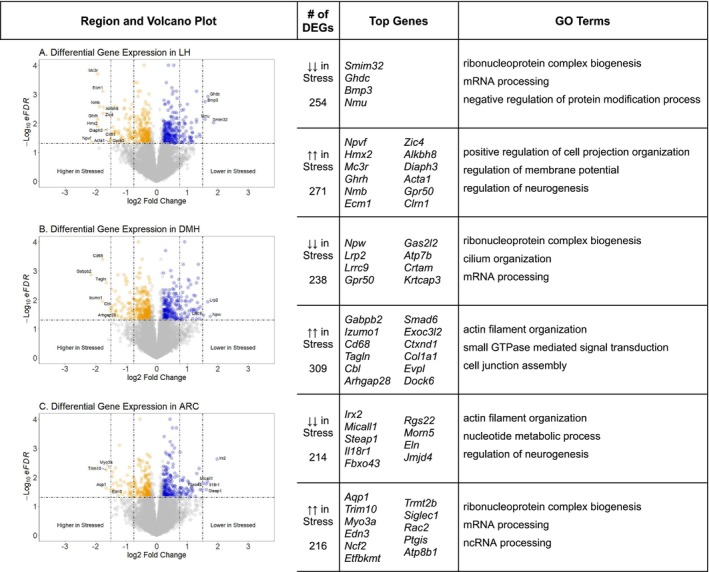
Results of differential gene expression (DEGs) in (A) lateral (LH), (B) dorsomedial (DMH) and (C) arcuate nucleus (ARC) subregions of the hypothalamus. Genes were determined to be differentially expressed if the log2 Fold Change was ≥|0.2| and significant (eFDR) <0.05. Top genes were identified as the greatest absolute value log2FC with an eFDR <0.05 (dashed horizontal line). Dashed vertical lines at |0.75| and |1.5| are included to illustrate numbers of genes at different ranges of change. ↑↑ indicates increased relative expression, ↓↓ indicates decreased relative expression. Top genes were selected by filtering the highest fold change while having an eFDR <0.05. Functional modules identified by Gene Ontology enrichment analysis (GO terms) are on the right.

In the LH, out of 11,354 genes, 271 genes had higher relative expression in Stressed subjects, and 254 genes had lower relative expression in Stressed subjects compared to Controls. In Figure 3A, volcano plots of gene expression and a selection of DEGs with the highest log 2‐fold change and lowest eFDR are highlighted, separated by increased and decreased relative expression. Values of log2FC and eFDR for top genes across all regions are reported in Table 1. Gene Ontology (GO) terms are also reported in Figure 3, displaying functional modules associated with clusters of highly differentially expressed genes. In the DMH, out of 11,350 genes, 309 genes had higher relative expression in Stress, and 238 genes had lower relative expression in Stress compared to Controls (Figure 3B). In the ARC, out of 11,444 genes, 216 genes had higher relative expression in Stress, and 214 genes had lower relative expression in Stress compared to Controls (Figure 3C). We also performed an overlap and incongruence analysis on DEGs, compiling genes that were up‐ or down‐expressed in multiple regions in Table 2, and genes that showed differential increased or decreased expression between multiple regions in Table 3. Only one gene, *Zfp36*, had increased expression across all 3 regions in Stressed subjects, and 33 other genes shared some overlap across multiple regions. Forty‐three genes showed incongruent differential expression, having higher relative transcript abundance in one region and lower abundance in another.

**TABLE 1:** Summary of top differentially expressed genes.

LH			DMH			ARC		
Gene	log2FC	eFDR	Gene	log2FC	eFDR	Gene	log2FC	eFDR
*Npvf*	−2.13038	0.0438	*Gabpb2*	−2.14412	0.0014	*Irx2*	1.978426	0.0024
*Hmx2*	−1.93924	0.013	*Izumo1*	−1.78528	0.0114	*Micall1*	1.660793	0.0142
*Mc3r*	−1.93781	0.0004	*Cd68*	−1.76592	0.0004	*Aqp1*	−1.65694	0.024
*Ghrh*	−1.93195	0.0074	*Npw*	1.758465	0.037	*Trim10*	−1.65321	0.0056
*Nmb*	−1.86515	0.0026	*Lrp2*	1.668749	0.0118	*Steap1*	1.627907	0.0266
*Smim32*	1.864586	0.0096	*Tagln*	−1.64991	0.0026	*Il18r1*	1.626486	0.0172
*Ecm1*	−1.75846	0.0008	*Lrrc9*	1.529791	0.034	*Myo3a*	−1.54983	0.0046
*Zic4*	−1.75188	0.0046	*Cbl*	−1.51526	0.0186	*Fbxo43*	1.53378	0.0164
*Alkbh8*	−1.73714	0.0028	*Arhgap28*	−1.50624	0.0246	*Edn3*	−1.52287	0.0268
*Ghdc*	1.679592	0.0012	*Smad6*	−1.48152	0.0044	*Rgs22*	1.487865	0.0272
*Diaph3*	−1.62275	0.0168	*Exoc3l2*	−1.47295	0.0204	*Ncf2*	−1.45757	0.0096
*Bmp3*	1.585725	0.0018	*Ctxnd1*	−1.45043	0.0164	*Etfbkmt*	−1.44306	0.0066
*Nmu*	1.575957	0.0072	*Gpr50*	1.429156	0.0176	*Trmt2b*	−1.43991	0.0272
*Acta1*	−1.53146	0.044	*Col1a1*	−1.42618	0.0312	*Morn5*	1.427706	0.027
*Gpr50*	−1.51998	0.0384	*Evpl*	−1.40366	0.0408	*Siglec1*	−1.42272	0.017
*Clrn1*	−1.51716	0.0364	*Gas2l2*	1.403227	0.045	*Rac2*	−1.4084	0.0288
*Krt77*	−1.46919	0.0246	*Atp7b*	1.367329	0.0006	*Eln*	1.391259	0.005
*Irs4*	−1.45883	0.0054	*Crtam*	1.357569	0.0202	*Jmjd4*	1.387232	0.017
*Isl1*	−1.43804	0.0234	*Krtcap3*	1.356831	0.0018	*Ptgis*	−1.34655	0.0048
*Slc7a14*	−1.43257	0.0136	*Dock6*	−1.35372	0.0148	*Atp8b1*	−1.3178	0.0154

*Note*: Summary of top highly differentially expressed genes across all regions analyzed. Top genes were selected by filtering the highest fold change while having an eFDR <0.05. Positive values indicate lower levels of transcript in Stressed subjects; negative values indicate higher levels in Stressed subjects. eFDR: enhanced false discovery rate, or permuted *p*‐value.

Abbreviations: ARC, arcuate nucleus of the hypothalamus; DMH, dorsomedial hypothalamus; LH, lateral hypothalamus; log2FC, log2 fold change in expression between groups.

**TABLE 2:** Differential gene expression region overlaps.

Gene	Direction	Regions	Description
*Zfp36*	Up	LH, DMH, ARC	Zinc finger protein 36
*Apcdd1*	Up	LH, DMH	Adenomatosis polyposis coli down‐regulated 1
*Armh3*	Up	LH, ARC	Armadillo‐like helical domain containing 3
*Clec2l*	Up	LH, DMH	C‐type lectin domain family 2, member L
*Cpne1*	Up	LH, ARC	Copine I
*Ebpl*	Up	LH, ARC	Emopamil binding protein‐like
*Ecpas*	Up	LH, ARC	Ecm29 proteasome adaptor and scaffold
*Entpd3*	Up	LH, DMH	Ectonucleoside triphosphate diphosphohydrolase 3
*Gpc1*	Up	LH, DMH	Glypican 1
*Ltbr*	Up	DMH, ARC	Lymphotoxin B receptor
*Mrpl3*	Up	DMH, ARC	Mitochondrial ribosomal protein L3
*Mtmr10*	Up	LH, DMH	Myotubularin related protein 10
*Nek8*	Up	LH, ARC	NIMA (never in mitosis gene a)‐related expressed kinase 8
*Npr1*	Up	LH, DMH	Natriuretic peptide receptor 1
*Sprn*	Up	LH, DMH	Shadow of prion protein
*Tchh*	Up	LH, ARC	Trichohyalin
*Angptl4*	Down	LH, ARC	Angiopoietin‐like 4
*Aopep*	Down	DMH, ARC	Aminopeptidase O
*Ccdc18*	Down	LH, DMH	Coiled‐coil domain containing 18
*Clk1*	Down	LH, DMH	CDC‐like kinase 1
*Fos*	Down	LH, ARC	FBJ osteosarcoma oncogene
*Hmgn5*	Down	LH, DMH	High‐mobility group nucleosome binding domain 5
*Icam2*	Down	LH, ARC	Intercellular adhesion molecule 2
*Med9*	Down	DMH, ARC	Mediator complex subunit 9
*Nek3*	Down	DMH, ARC	NIMA (never in mitosis gene a)‐related expressed kinase 3
*Nt5c2*	Down	LH, DMH	5′‐Nucleotidase, cytosolic II
*Parp12*	Down	LH, ARC	Poly (ADP‐ribose) polymerase family, member 12
*Pex12*	Down	LH, DMH	Peroxisomal biogenesis factor 12
*Rad23a*	Down	LH, DMH	RAD23 homolog A, nucleotide excision repair protein
*Rspry1*	Down	DMH, ARC	Ring finger and SPRY domain containing 1
*Smim15*	Down	LH, DMH	Small integral membrane protein 15
*Steap1*	Down	DMH, ARC	Six transmembrane epithelial antigen of the prostate 1
*Tent5a*	Down	DMH, ARC	Terminal nucleotidyltransferase 5A
*Tut1*	Down	DMH, ARC	Terminal uridylyl transferase 1, U6 snRNA‐specific

*Note*: Differential gene expression overlap analysis. Direction indicates if gene transcripts increased or decreased in relative abundance in socially stressed hamsters and in which regions.

**TABLE 3:** Differential gene expression region incongruence.

Gene	Region up	Region down	Description
*Tchh*	LH, ARC	DMH	Trichohyalin
*Abhd14a*	LH	ARC	Abhydrolase domain containing 14A
*Btg1*	LH	DMH	BTG anti‐proliferation factor 1
*Cckar*	LH	DMH	Cholecystokinin A receptor
*Efl1*	LH	DMH	Elongation factor like GTPase 1
*Gjc1*	LH	ARC	Gap junction protein, gamma 1
*Gpr50*	LH	DMH	G‐protein‐coupled receptor 50
*Gtf2h3*	LH	ARC	General transcription factor IIH, polypeptide 3
*Hdac4*	LH	DMH	Histone deacetylase 4
*Htr1b*	LH	DMH	5‐Hydroxytryptamine (serotonin) receptor 1B
*Ift88*	LH	DMH	Intraflagellar transport 88
*Irs4*	LH	DMH	Insulin receptor substrate 4
*Med24*	LH	ARC	Mediator complex subunit 24
*Mustn1*	LH	ARC	Musculoskeletal, embryonic nuclear protein 1
*Nol12*	LH	DMH	Nucleolar protein 12
*Nsun7*	LH	DMH	NOL1/NOP2/Sun domain family, member 7
*Pdyn*	LH	DMH	Prodynorphin
*Rcc1l*	LH	DMH	Reculator of chromosome condensation 1 like
*Riox1*	LH	ARC	Ribosomal oxygenase 1
*Slc16a11*	LH	ARC	Solute carrier family 16 (monocarboxylic acid transporters), member 11
*Slc7a14*	LH	DMH	Solute carrier family 7 (cationic amino acid transporter, y + system), member 14
*Vps18*	LH	ARC	VPS18 CORVET/HOPS core subunit
*Desi1*	DMH	LH	Desumoylating isopeptidase 1
*Dut*	DMH	LH	Deoxyuridine triphosphatase
*Fkbp15*	DMH	LH	FK506 binding protein 15
*Gpr146*	DMH	ARC	G protein‐coupled receptor 146
*Irx2*	DMH	ARC	Iroquois homeobox 2
*Mid1ip1*	DMH	ARC	Mid1 interacting protein 1 (gastrulation specific G12‐like (zebrafish))
*Mss51*	DMH	LH	MSS51 mitochondrial translational activator
*Mtg2*	DMH	LH	Mitochondrial ribosome associated GTPase 2
*Pex7*	DMH	ARC	Peroxisomal biogenesis factor 7
*Sidt1*	DMH	LH	SID1 transmembrane family, member 1
*Skida1*	DMH	LH	SKI/DACH domain containing 1
*Slc9a3r2*	DMH	LH	Solute carrier family 9 (sodium/hydrogen exchanger), member 3 regulator 2
*Smad6*	DMH	ARC	SMAD family member 6
*Tmc7*	DMH	LH	Transmembrane channel‐like gene family 7
*Tph2*	DMH	ARC	Tryptophan hydroxylase 2
*Bmp3*	ARC	LH	Bone morphogenetic protein 3
*Fnbp4*	ARC	DMH	Formin binding protein 4
*Iws1*	ARC	DMH	IWS1, SUPT6 interacting protein
*Snrpb2*	ARC	DMH	U2 small nuclear ribonucleoprotein B
*Stim2*	ARC	DMH	Stromal interaction molecule 2
*Zcchc8*	ARC	DMH	Zinc finger, CCHC domain containing 8

*Note*: Differential gene expression incongruence analysis. First organized by which regions conflicting genes were increased in expression in stressed subjects, then which regions they had decreased relative expression in.

### 3.3 Highly differentially expressed genes do not correlate with subjugation behaviors

None of the correlations between stress behaviors and top 10 differentially expressed genes were significantly different (Figure S3). However, the endothelin‐3 gene *edn3* had the strongest correlations observed, and r values were high in comparison to all three behaviors: average attacks (*r* = 0.689, unadjusted *p* = .028, Holm‐adjusted *p* = 1, *n* = 10), tail‐ups (*r* = −0.789, unadjusted *p* = .007, Holm‐adjusted *p* = .43, *n* = 10), resident scent‐markings (*r* = 0.745, unadjusted *p* = .014, Holm‐adjusted *p* = .84, *n* = 10).

### 3.4 WGCNA reveals co‐expressed gene modules uniquely associated with social stress

**FIGURE 4:**
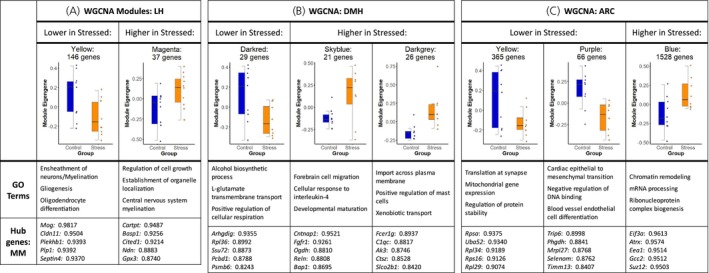
Results of weighted gene coexpression network analysis (WGCNA) in (A) lateral (LH), (B) dorsomedial (DMH) and (C) arcuate nucleus (ARC) subregions of the hypothalamus. All noted modules are significantly differentially expressed between groups, with boxplots of module eigengene values shown per region. Below are Gene Ontology (GO) terms associated with differentially expressed modules and hub genes with the highest module membership (MM).

Figure S2 summarizes all WGCNA modules identified. Modules differentially expressed between groups are highlighted in Figure 4, including the GO terms most associated with differentially expressed modules. Genes with high module membership (MM—genes that are most highly correlated with other genes in the module) are also listed. In the LH, WGCNA identified 14 modules of highly correlated genes. Two of these (yellow and magenta) exhibited significant differences in ME expression between groups (Figure 4A). The yellow module (*b* = −0.224 ± 0.097, *p* < .05) was most strongly associated with myelination and had ME scores that were significantly decreased in the LH. The magenta module (*b* = 0.212 ± 0.099, *p* < .05) was also associated with myelination processes along with cell growth and organization and was increased in stressed individuals.

In the DMH, WGCNA identified 31 modules. Three of these (darkred, skyblue, and darkgrey) were differentially expressed between groups (Figure 4B). The darkred module (*b* = −0.251 ± 0.107, *p* < .05) was associated with alcohol‐related bioprocesses and glutamate transport and was decreased with stress. The skyblue module (*b* = 0.254 ± 0.107, *p* < .05) was associated with neuronal development and immune function and was increased in stress. The darkgrey module (*b* = 0.315 ± 0.095, *p* < .01) was also increased in stress and was also associated with membrane transport and immune function.

In the ARC, WGCNA identified 11 modules. Three of these (yellow, purple, and blue) were differentially expressed between groups (Figure 4C). The yellow module (*b* = −0.235 ± 0.110, *p* < .05) was associated with synaptic function and cellular energy use and was lower in Stressed subjects. The purple module (*b* = −0.321 ± 0.095, *p* < .01) was also lower with Stress and was associated with DNA processing and blood vessel function. The blue module (*b* = 0.262 ± 0.106, *p* < .05) was increased in Stressed subjects and was associated with mRNA processing and epigenetic functions.

## 4 DISCUSSION

Stressed hamsters gained weight at an increased rate, had higher food efficiency, and tended to have more body fat than controls, supporting prior work in this model.^26^ Overall, approximately 500 genes were differentially expressed between groups in each region sampled. In the present analysis, we focused on hypothalamic regions functionally associated with appetite and other metabolic processes. We observed changes in the expression of a number of orexigenic transcripts across regions. Due to prior research in the lab, we had a particular interest in LH orexin/hypocretin, expecting its expression to be elevated in stressed subjects that gain more weight than controls. Interestingly, there was only a trend that LH orexin gene expression (*hcrt*) was increased in stressed subjects. This was not necessarily surprising, given prior results from our lab that suggest stress‐induced differences in orexin are not at the innervation level, but more likely caused by downstream receptor changes or changes to other orexin neuron action potential firing mechanisms, such as upstream inputs or intrinsic traits like resting membrane potential.^28^ However, growth hormone releasing hormone (*ghrh*) transcript was increased in the LH of stressed subjects. It was recently reported that activation of growth hormone receptors in LH orexin neurons promotes their cellular and related behavioral functions,^67^ which could be related to a change in orexin firing, if not orexin expression or innervation. Additionally, the LH WGCNA magenta module that was increased in expression in stressed subjects was also related to cellular growth. Neuromedin U transcript was also decreased in the LH. In rats, this neuropeptide is typically expressed in the arcuate nucleus and has an anorexic influence,^68^ making its possible downregulation in our enhanced eaters unsurprising. Decreased NMU transcript may reflect a compensatory mechanism, as NMU administration induces stress‐response behaviors such as self‐grooming in rats and mice.^69^ Similarly, neuropeptide W (NPW) transcript was decreased in the DMH. NPW activity inhibits food intake and stimulates thermogenesis.^70^ Interestingly, NPW is widely involved in other stress, growth, and sex hormone‐related processes, making it an excellent candidate for further study in this model.^71^, ^72^

Stressed hamsters also had increased relative transcript of Neuropeptide VF precursor (*Npvf*) RNA in the LH. *Npvf* encodes a precursor peptide for RFRP‐3, which makes the increase in the LH and absence of change in the DMH interesting. In hamsters, the occurrence of RFRP‐3 expressing cell bodies can extend to perifornical areas.^73^ While the majority of DMH *Npvf*‐expressing cells did not differ between groups, a subset of these more laterally located neurons has increased expression due to stress. Nevertheless, the possible upregulation of RFRP‐3, the formerly putative gonadotropin inhibitory hormone,^36^, ^73^, ^74^ aligns with prior findings of short‐term, 50% reductions in testosterone in stressed male hamsters.^37^ Stressed adolescent male hamsters also tend to hoard more food,^26^, ^27^ and RFRP‐3 is involved in food hoarding behavior in hamsters, though more so in low estrogen‐state females.^75^ Additionally, in mice, whole hypothalamic *Npvf* expression is decreased in cold temperatures alongside enhanced thermogenesis, regardless of diet, leptin, or body fat,^76^ suggesting it may have a metabolic role independent of RFRP‐3 that yet functionally coordinates with its gonadotropin inhibitory role. Increased *Npvf* transcript may also alter sleep patterns, as *Npvf* expression is necessary for sleep in zebrafish.^77^ Here, *Npvf* may be playing competing roles regarding metabolic processes that require further study.

In the LH, the melanocortin 3 receptor transcript was significantly increased in stressed subjects. *Mc3r* codes for a receptor of melanocyte‐stimulating hormone, which has anorexigenic downstream effects.^78^, ^79^ Since the LH is also the home of α‐MSH containing neurons,^80^, ^81^ increased MC3R RNA suggests a local action altered by stress, potentially in competing homeostatic processes.

We also observed differential up‐ and down‐expression of the orphan receptor G protein‐coupled receptor 50 (*Gpr50*) transcript between the LH and DMH. GPR50 is involved in cellular leptin sensitivity and leptin‐induced thermogenesis in mice.^82^, ^83^, ^84^ These processes are likely altered by stress in hamsters, both in relation to body fat gain and a number of other differentially expressed genes. We observed no *Gpr50* expression in the ARC (which very likely included tissue from the median eminence due to their proximity) despite consistent expression in the median eminence in other rodents and humans,^84^ which could be an artifact of sampling. GPR50 is also necessary for cellular stress‐induced mitochondrial autophagy responses,^85^ deepening its metabolic role. Along this line, the DMH WGCNA darkred module, which is involved in cellular respiration, was also decreased in expression in stressed subjects. The five most central hub genes in this module have few functions classically associated with stress exposure, emphasizing the importance of analyzing transcriptomic datasets from more than just a DEG perspective, and highlighting these genes as potential focal points of future studies. Additionally, *selenom*, a hub gene in the lower‐expression ARC WGCNA purple module, has also been previously associated with the development of insulin resistance, obesity, and neuroinflammation when it is exogenously inhibited or knocked out.^86^, ^87^ While others have investigated its role in the hippocampus^87^ and adipose tissue,^86^ its role in the hypothalamus is a potential target of future research.

Other genes in the lower‐expression ARC WGCNA purple module and the DMH darkred module are associated with ribosomal and mitochondrial ribosomal proteins (*Rps*, *Rpl*, and *Mrpl*). However, Gene Ontology terms derived from differential gene expression suggest opposing patterns. In the ARC, one GO term that was higher in expression in stressed subjects was ribonucleoprotein complex biogenesis, while the same GO term was significantly decreased in expression in the LH and DMH in stressed subjects. Others have found that chronic social stress increases many of these ribosomal genes in whole‐hypothalamus RNAseq in C57BL/6J mice.^88^ Our findings clearly show there is more nuance to these mechanisms, requiring further study.

We observed a number of transcriptomic changes supporting our hypothesis that stress induces alterations to development‐related gene expression. Across regions, a variety of both enhanced and decreased GO terms, WGCNA modules, and differentially expressed genes were related to RNA processing, development, neurogenesis, and maturation. As these data are from adolescent subjects, changes in development‐related processes is not surprising. In fact, these changes could reflect longer‐term alterations in gene expression. For example, in mice, similar developmental modules, such as those involved in multicellular organism development, nervous system development, and cell differentiation, are altered in adult animals that were exposed to early life stress,^89^ suggesting that these changes can be quite long‐lasting. Nevertheless, a primary GO term related to highly DEGs, mRNA processing, was decreased in stressed hamsters in the LH and DMH, but increased in the ARC. This somewhat aligns with WGCNA‐derived modules in these regions. The ARC WGCNA blue module was increased and contributes to RNA processing. Many of the hub genes in this module are directly related to chromatin remodeling, which is well known to be altered by stress,^90^ and suggests potentially long‐term epigenetic changes in this model. Despite these changes, it is interesting that we observed a significant increase of growth hormone releasing hormone transcript (*ghrh*) in the LH, but not in other hypothalamic regions.

WGCNA highlighted modules related to myelination both increased (LH magenta) and decreased (LH yellow) by stress in the LH. Others have shown links between social dominance behaviors and myelination. In mice exposed to a shift in social environment that retained a prior dominant status in a previous colony, social reorganization was associated with myelination and oligodendrocyte development genes and modules in the amygdala.^91^ Direct chronic social defeat in mice also decreases myelination‐related gene expression, followed by a reduction in oligodendrocyte number and myelin protein levels and thickness in the medial prefrontal cortex, hippocampus, and nucleus accumbens,^92^, ^93^ though there is some variability in the prefrontal cortex and basolateral amygdala.^94^, ^95^ Our results are interesting in conjunction with the fact that adolescent‐stressed hamsters become more socially aggressive, suggesting that myelinating processes may play a broader role in response to stress and social behaviors. Furthermore, rats that experienced chronic social stressors from mid‐ to late‐adolescence have altered hippocampal white matter structure, especially paired with obesogenic dietary conditions.^96^ In humans that experienced early life abuse, these traits are correlated, such that in abused individuals, increased BMI is associated with decreased forebrain white matter connectivity, particularly with the lateral hypothalamus.^97^ Hub genes associated with these myelination‐related modules included *mog*, myelin oligodendrocyte glycoprotein, and *cartpt*, cocaine and amphetamine regulated transcript (CART) preprotranscript. While the role of *mog* in response to stress is less understood, other studies have found that *cartpt* expression is positively correlated with stress susceptibility in the hippocampus,^98^ but positively associated with stress resilience in the anterior cingulate.^99^ The fact that we observed no differential expression of *cartpt* transcript despite its role in a functionally altered WGCNA module suggests increased study of CART in the LH is warranted. Thus, the effects of stress on myelinating processes and their interaction with metabolic function are important for continued research.

Gene expression in our ARC samples was not as expected in some aspects. While others have found that adolescent stress in rats resulted in reduced *Pomc* and *Cartpt* expression in the ARC alongside a reduction in body weight,^100^ we observed no differences in expression of the classic appetite‐related ARC systems such as NPY/AgRP or POMC/CART. Though it is interesting that we observed no changes in these systems, this species difference in gene expression may align with species‐typical metabolic outcomes.^101^ While we did observe differential expression in over 400 genes in the ARC, many of them, such as *Aqp1*, are involved in ventricle function^102^ or immune function, such as *Ncf2* or *Crlf1*.^103^, ^104^ WGCNA also highlighted the purple module in the ARC, related to blood vessel development, which was lower in stressed subjects. These processes may be related to stress and obesity through the neuroinflammatory effects of both of these conditions,^96^ thus causing changes in information received by metabolic‐sensory arcuate neurons instead of gross changes in their expression. Follow‐up studies are needed to investigate this further. One of the decreased GO terms derived from highly DEGs in the ARC was related to actin filament organization, further suggesting impairment of mechanical cellular sensory mechanisms.

More broadly, a plasma membrane permeability‐related module in the DMH (darkgrey) was increased in stressed subjects. One hub gene in this module, *c1qc*, encodes the complement C1q C chain. *C1qc* is a component of the complement cascade, relevant for proper immune function. Interestingly, increased expression of *c1qc*‐related proteins in individuals that experienced trauma at age 12 was positively correlated with the likelihood of psychotic experiences at age 18.^105^ Furthermore, *c1qc* transcript is increased in atherosclerotic arteries.^106^ Therefore, both behaviorally and physiologically, elements of the complement cascade warrant further study in the stressed hamster model. The DMH WGCNA skyblue module was also related to immune function, specifically cellular response to interleukin‐4. Hub genes here included *fgfr1*, fibroblast growth factor receptor. *Fgfr1* has been more classically associated with obesogenic states in adipocytes,^107^, ^108^ and other fibroblast growth factor mechanisms are involved in the neural response to stress hormones.^109^ Finally, the increased *zfp36* transcript across all regions sampled in stressed subjects is notable. *Zfp36*, zinc finger protein 36, is also involved in immune‐inflammation responses and could be a critical hypothalamic target of future study.^110^

Finally, while there were no statistically significant correlations between behaviors observed during resident‐intruder interactions, expression levels of the endothelin gene *Edn3* did have strong relationships with attacks received, tail‐ups displayed, and scent markings made by the resident, and were significant pre‐adjustment. Endothelin‐1 microinfusion stimulates ghrelin release,^111^ which could be related to stress‐induced appetite increases. Endothelin‐1 and ‐3 may also play protective roles against neuroinflammation caused by changes in diet.^112^ Prior research has rarely found correlations between the resident‐intruder behaviors and most other metrics.^24^, ^26^, ^27^, ^28^ However, the present relationship with most of the subjugation behaviors warrants future follow‐up, as there are fewer stress‐related studies on endothelin‐3 than endothelin‐1.

It must be stated that the hypothalamus is a highly heterogeneous region containing many cell types. For example, the differences observed in *Gpr50* expression in the DMH could also be a sampling artifact due to its proximity to the 3rd ventricle, as the tanycytes in this region highly express *Gpr50* as well.^84^ Furthermore, Tag‐seq is a bulk RNA sequencing technique and is not able to identify sources of cell types related to any RNA's expression level. This limits the types of direct conclusions we would be able to make from the present data, though it is certainly useful in generating hypotheses for future experiments.

Overall, the study outcome supported our initial hypotheses that a variety of genes and functional genetic modules related to metabolic processes and development were altered by stress exposure. While the focus was on appetite‐ and obesity‐related genes, it is clear that the experience causes profound alterations in multiple hypothalamus‐related systems. The present data highlight systems related to myelination and immune function in the brain from multiple analytical perspectives as potential drivers of stress‐induced changes. Changes in myelination could relate to differences in motivated or social behaviors observed in this model. Altered immune function may impact metabolic function. The present data are useful in driving more specific, targeted hypothesis‐based approaches in further research. Future studies will likely further relate our findings to the behavioral and metabolic consequences of social stress during adolescence, whether they result in short‐ or long‐term effects.

## AUTHOR CONTRIBUTIONS

**Kevin M. Moran:** Conceptualization; investigation; writing – original draft; methodology; validation; writing – review and editing; formal analysis; data curation; visualization. **Tyler M. Milewski:** Methodology; formal analysis; writing – review and editing; validation; visualization. **James P. Curley:** Funding acquisition; conceptualization; writing – review and editing; methodology; supervision; resources; project administration. **Yvon Delville:** Conceptualization; funding acquisition; writing – review and editing; methodology; supervision; resources; project administration.

## CONFLICT OF INTEREST STATEMENT

The authors declare no conflicts of interest.

## PEER REVIEW

The peer review history for this article is available at https://www.webofscience.com/api/gateway/wos/peer‐review/10.1111/jne.70067.

## Supporting information


**Figure S1.** Principal component analysis by region of interest. Separation of normalized gene expression data of samples by region.

**Figure S2.** Results of weighted gene coexpression network analysis (WGCNA) in (A) lateral (LH), (B) dorsomedial (DMH) and (C) arcuate nucleus (ARC) subregions of the hypothalamus. Presented are each module and the number of genes in that module. Values in the table represent the Pearson correlation interaction term between Control and Stressed groups, with asterisks marking statistically significant differences: *p < .05, **p < .01. Positive values are higher expression of the module in the Stress group, negative values are lower expression in the Stress group.

**Figure S3.** Spearman correlation between top differentially expressed genes and subjugation behaviors. Behaviors recorded during social stress were correlated with the top 10 most differentially expressed genes (the five genes with the highest positive and negative relative expression, determined by highest absolute log2FC and eFDR <0.05). Only average attacks received by the intruder, tail‐ups displayed by the intruder, and scent markings from the resident were correlated, as the other behaviors occurred with too little frequency for valid correlation. All comparisons were made with Spearman correlation (with r values displayed in table), as gene expression data was not normally distributed, and p values were Holm‐corrected for multiple comparisons. No comparisons had a corrected *p* < .1.

## Data Availability

The data that support the findings of this study are openly available in Series GSE289511 at https://www.ncbi.nlm.nih.gov/geo/query/acc.cgi?acc=GSE289511. Raw data will be released early on request of reviewers and after publication. Analysis pipeline github repository: https://github.com/kmoran10/Project-RNA-tag-seq.
