# Supplementary figures and images for: Stress alters hypothalamic gene expression in adolescent male Golden hamsters
Source: J Neuroendocrinol. 2025 Jul 14;37(9):e70067. doi: 10.1111/jne.70067 (PMC12404909; doi:10.1111/jne.70067)

PCA Grouping by Region of Interest

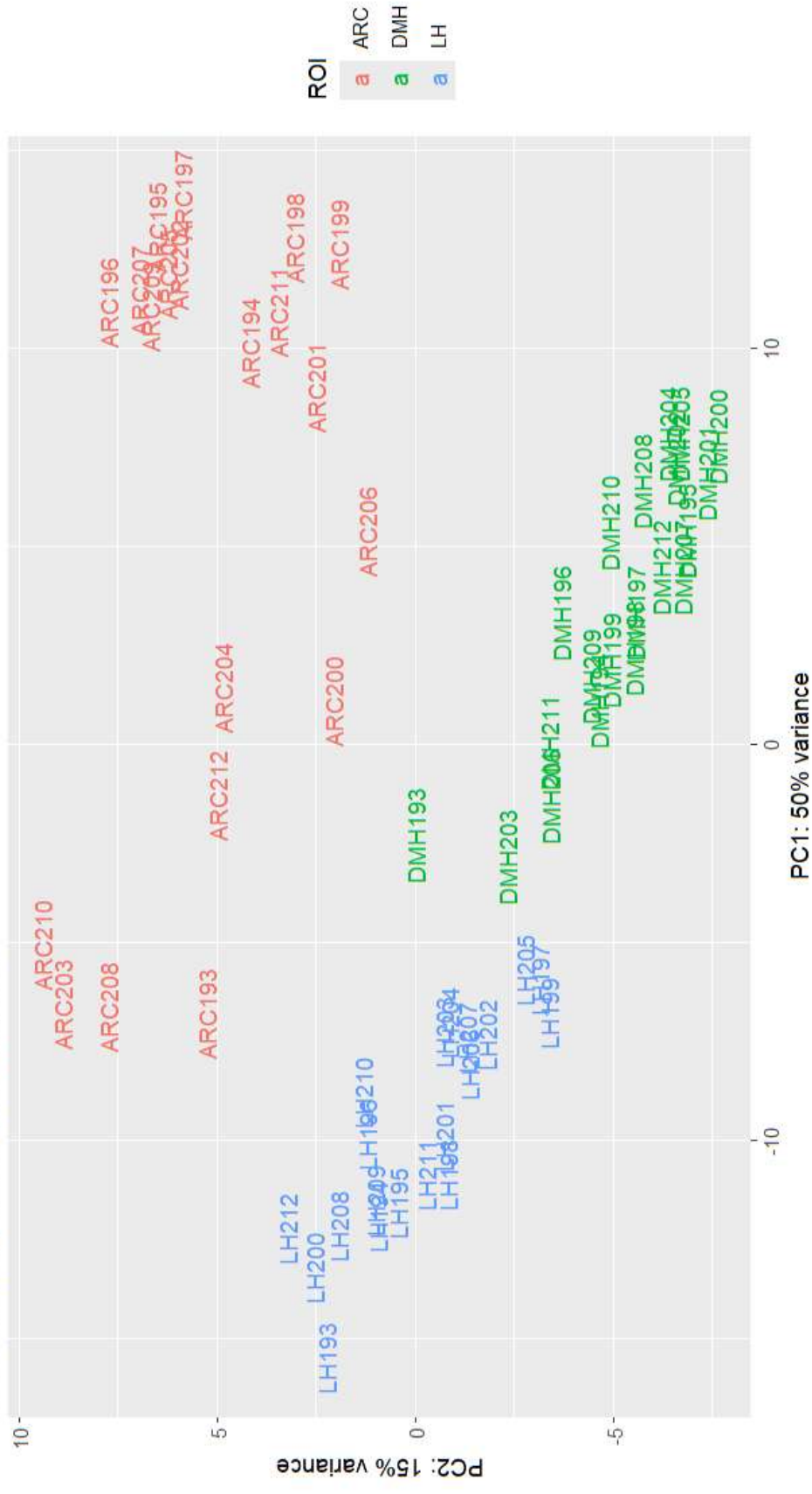

Supplement: Supplementary file 1 — Figure S1. Principal component analysis by region of interest. Separation of normalized gene expression data of samples by region. [file JNE-37-e70067-s003.pdf]
