# Supplementary material for: Stress alters hypothalamic gene expression in adolescent male Golden hamsters
Source: J Neuroendocrinol. 2025 Jul 14;37(9):e70067. doi: 10.1111/jne.70067 (PMC12404909; doi:10.1111/jne.70067)
Supplement: Supplementary file 2 — Figure S2. Results of weighted gene coexpression network analysis (WGCNA) in (A) lateral (LH), (B) dorsomedial (DMH) and (C) arcuate nucleus (ARC) subregions of the hypothalamus. Presented are each module and the number of genes in that module. Values in the table represent the Pearson correlation interaction term between Control and Stressed groups, with asterisks marking statistically significant differences: *p < .05, **p < .01. Positive values are higher expression of the module in the Stress group, negative values are lower expression in the Stress group. [file JNE-37-e70067-s001.pdf]

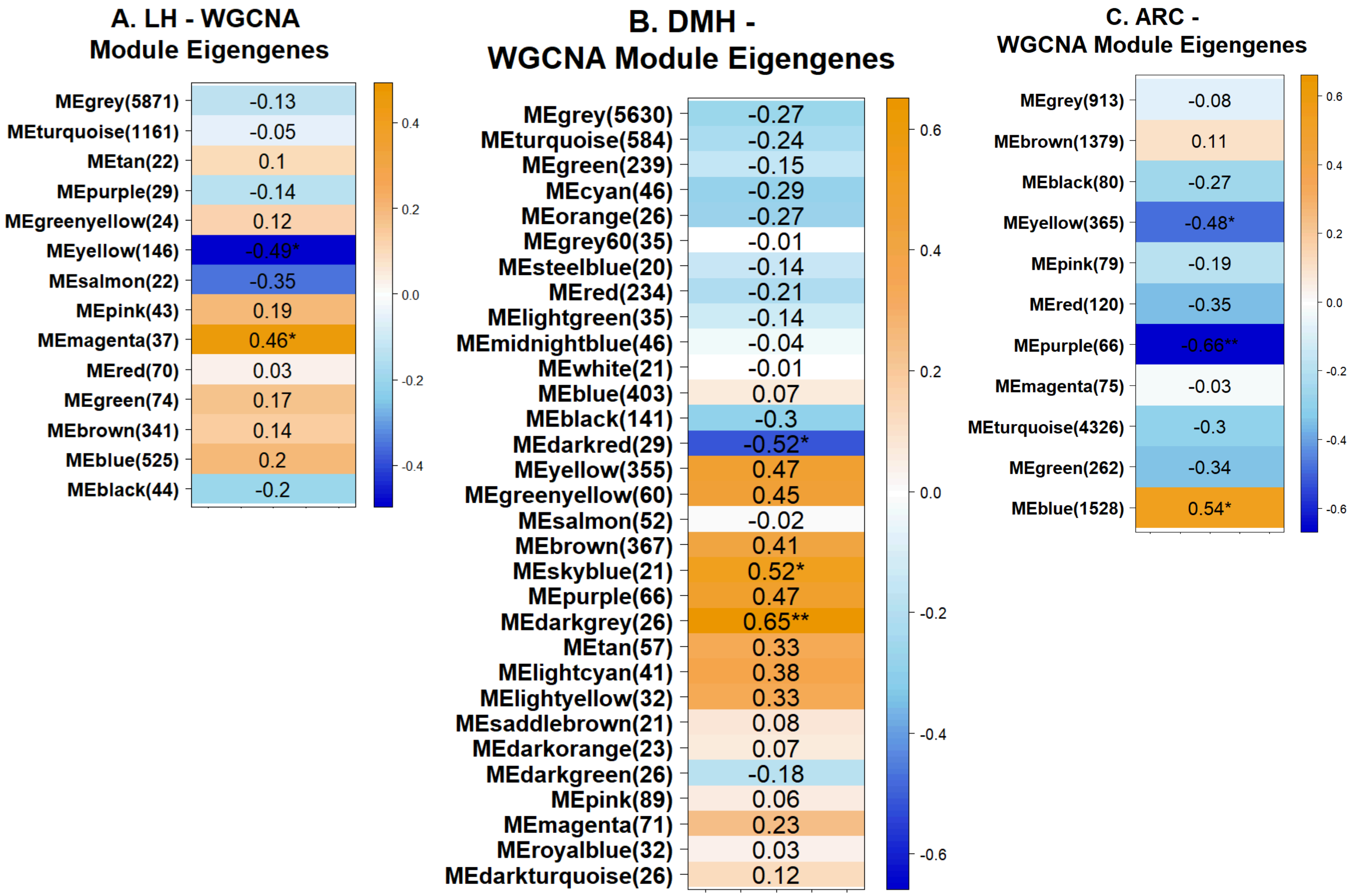


Figure S2. Results of weighted gene coexpression network analysis (WGCNA) in A. lateral (LH), B. dorsomedial (DMH) and C. arcuate nucleus (ARC) subregions of the hypothalamus. Presented are each module and the number of genes in that module. Values in the table represent the Pearson correlation interaction term between Control and Stressed groups, with asterisks marking statistically significant differences: * = p < 0.05, ** = p < 0.01. Positive values are higher expression of the module in the Stress group, negative values are lower expression in the Stress group.
