# Supplementary material for: Stress alters hypothalamic gene expression in adolescent male Golden hamsters
Source: J Neuroendocrinol. 2025 Jul 14;37(9):e70067. doi: 10.1111/jne.70067 (PMC12404909; doi:10.1111/jne.70067)

A. Top LH genes and Subjugation Correlations (Holm-corrected)

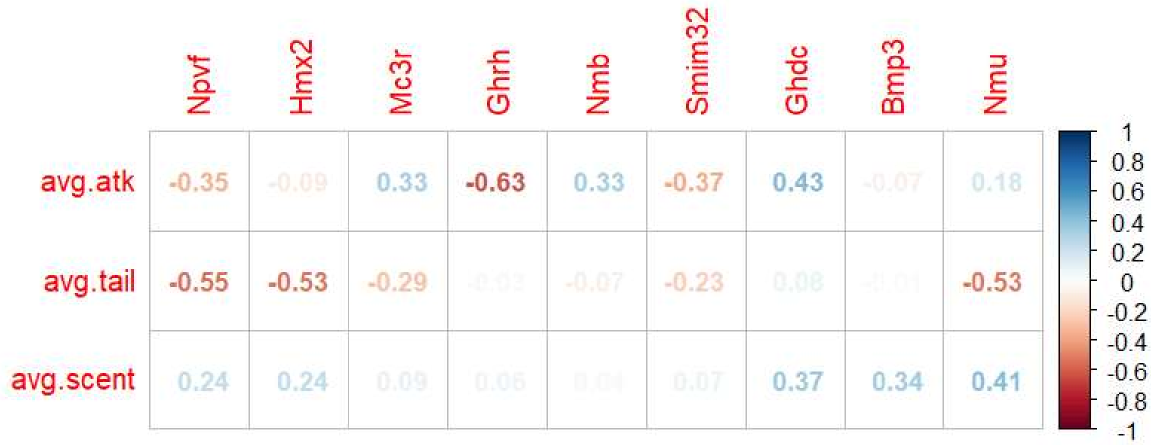

B. Top DMH genes and Subjugation Correlations (Holm-corrected)

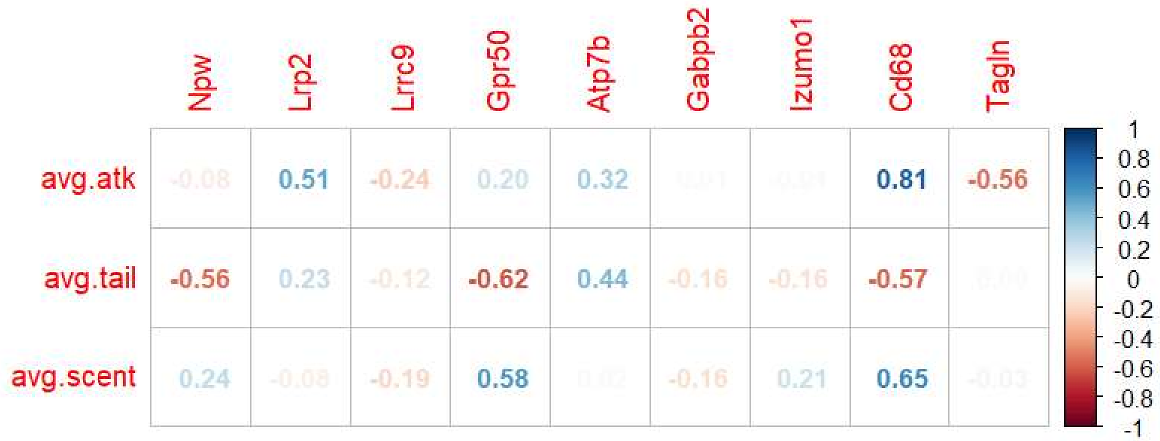

C. Top ARC genes and Subjugation Correlations (Holm-corrected)

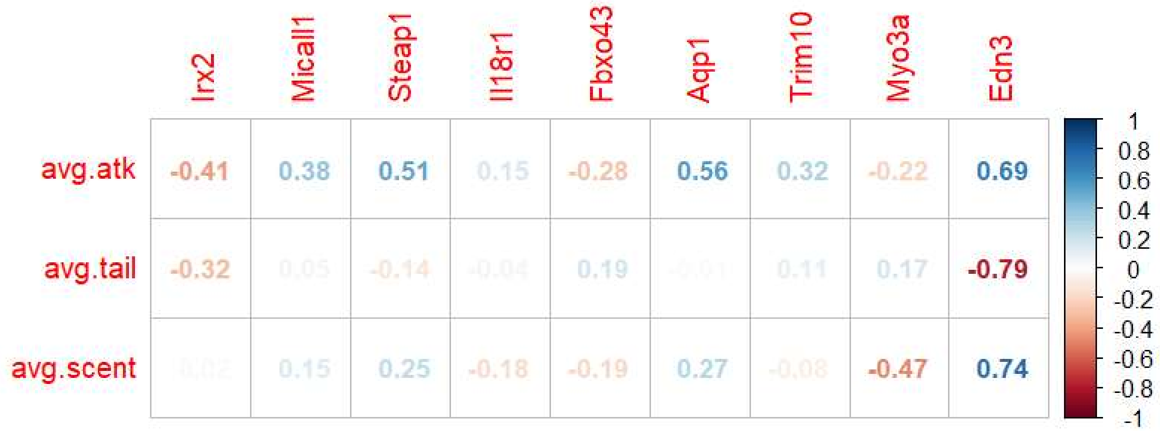

Supplement: Supplementary file 3 — Figure S3. Spearman correlation between top differentially expressed genes and subjugation behaviors. Behaviors recorded during social stress were correlated with the top 10 most differentially expressed genes (the five genes with the highest positive and negative relative expression, determined by highest absolute log2FC and eFDR <0.05). Only average attacks received by the intruder, tail‐ups displayed by the intruder, and scent markings from the resident were correlated, as the other behaviors occurred with too little frequency for valid correlation. All comparisons were made with Spearman correlation (with r values displayed in table), as gene expression data was not normally distributed, and p values were Holm‐corrected for multiple comparisons. No comparisons had a corrected p < .1. [file JNE-37-e70067-s002.pdf]
